# Peer Review of “Sexual Health Assessment Is Vital to Whole Health Models of Care”

**DOI:** 10.2196/39927

**Published:** 2022-07-28

**Authors:** Cynthia Darling-Fisher

**Affiliations:** 1 School of Nursing University of Michigan Ann Arbor, MI United States

**Keywords:** sexual health, sexual health assessment, veteran, health equity, health assessment, whole health model, communication, communication barrier, technological barrier, health care, sexuality, sexual orientation, gender identity, sex, gender, model, care, barrier, well-being, comfort, assessment, EHR, electronic health record, quality, equity

*This is a peer-review report submitted for the paper “Sexual Health Assessment Is Vital to Whole Health Models of Care.”*

## Round 1 Review

### General Comments

This is an excellent paper [1] that should be published. It’s well-written, informative, and important to disseminate to the health care community. This paper addresses an initiative by the Veterans Affairs (VA) health care system to address an issue important for providing high-quality comprehensive patient care. It is great that addressing sexual health assessment in their electronic health record (EHR) is being implemented by the VA since federal programs often influence national implementation. The additions presented in this paper need to be incorporated into other EHR programs, like Epic. Integration of detailed sexual health information in patient documentation is important to address preventive care, promote healthy sexual functioning, and optimize overall health and well-being. Having done research on the use of surveys to address sensitive topics such as sexual health and sexually transmitted infection risk, I found that patients of all ages are very willing to answer honestly (and in detail) about their sexual health on surveys, which then provides a time-efficient and useful way to open this discussion. This approach also allows health care providers to introduce these sensitive topics and, when patients are willing, provide health education, health promotion, and appropriate treatment when needed. I have also used this approach in my own clinical practice. This use of surveys to obtain and open the discussion on sensitive health issues has been documented in numerous research projects. It has also been shown to promote patient satisfaction with their care since it increases their sense of “being heard” by their providers. It also allows providers to provide more accurate care.

This paper is important to disseminate information about how a major health system has recognized the importance of sexual health assessment and found a way to implement this and incorporate it into their EHR. It also highlights the need to educate providers to orient them to the new system and provides ways to do this to assist them in better approaching sensitive health issues.

### Specific Comments

Great information about the VA’s approach. A particular strength of this paper is the presentation of prompts at different levels of assessment for patients who are hospitalized and more comprehensive visits.

#### Major Comments

1. Excellent background information

2. The pocket card format is excellent and serves several purposes: makes it easy for providers to follow a template and validates the importance of this information in providing patient care, and the repetition of obtaining this data in practice with all patients reinforces the importance of obtaining this data and can reduce provider resistance to asking these questions.

3. Following the pocket card questions (comprehensive form) nicely addresses and includes the patient’s partner information in the history. This reinforces for the provider the benefit of obtaining this information. The fact that the provider/EHR asks for partner information also highlights for the patient the importance to consider their partner(s) when addressing their own sexual health. This can also be a great trigger for patient education, which moves beyond the basic “plumbing” aspect of sexual health information (eg, how things work).

#### Minor Comments

4. A minor suggestion would be to provide a little more detail about provider education in the implementation of this new format. This could include role-playing with debriefing to help providers address their own concerns/reluctance to talk about sexual issues with their patients.

## Abbreviations

EHR: electronic health record
VA: Veterans Affairs
